# Epidemiological Study on Cutaneous Leishmaniasis in an Endemic Area, of Qom Province, Central Iran

**Published:** 2017-09-08

**Authors:** Abedin Saghafipour, Hassan Vatandoost, Ali Reza Zahraei-Ramazani, Mohammad Reza Yaghoobi-Ershadi, Moharram Karami Jooshin, Yavar Rassi, Mohammad Reza Shirzadi, Amir Ahmad Akhavan, Ahmad Ali Hanafi-Bojd

**Affiliations:** 1 Department of Medical Entomology and Vector Control, School of Public Health, International Campus (IC-TUMS), Tehran University of Medical Sciences, Tehran, Iran; 2 Department of Medical Entomology and Vector Control, School of Public Health, Tehran University of Medical Sciences, Tehran, Iran; 3 Department of Environmental Chemical Pollutants and Pesticides, Institute for Environmental Research, Tehran University of Medical Sciences, Tehran, Iran; 4 Qom Provincial Health Center, Qom University of Medical Sciences, Qom, Iran; 5 Communicable Diseases Management Center, Ministry of Health and Medical Education, Tehran, Iran

**Keywords:** Zoonotic cutaneous leishmaniasis, *Leishmania major*, *Phlebotomus papatasi*, ITS1-PCR, Iran

## Abstract

**Background:**

Cutaneous leishmaniasis (CL) is one of the most important health problems in many areas of Iran. There are two forms of the disease in Iran, anthroponotic and zoonotic CL. This study conducted to assess the epidemiological situation of CL in an endemic area of Qom Province, central Iran from Apr to Nov 2015.

**Methods:**

The sticky paper traps and aspirating tubes were used for collecting adult sand flies. Sherman traps and small insect nets were used to capture rodents and small mammals. Giemsa staining was used for preparing the expanded smear and followed by PCR for identifying the causative agent in human, vectors, and reservoirs. In this study, relative frequency of CL was also calculated.

**Results:**

Fourteen species of Phlebotomine sand flies were collected. *Phlebotomus papatasi* (61.74%) was the predominant species through the period of activity. Overall, 62 *Meriones libycus*, 8 *Nesokia indica*, 4 *Mus musculus*, 16 *Allactaga elater* and 2 *Hemiechinus auritis* were caught. PCR technique showed 6 out of 150 *P. papatasi* (2%), two out of 62 *M. libycus* (3.23%) and all of suspected human’s skin tissue samples (100%) were infected with *Leishmania major*. The relative frequency of CL was 0.30%.

**Conclusion:**

This is the first detection of *L. major* within *P. papatasi*, *M. libycus* and human in Kahak District in Qom Province of Iran. Zoonotic cycle of CL exists in this area, *L. major* is the causative agent, *P. papatasi* is the main vector and *M. libycus* is the main reservoir of the disease.

## Introduction

Currently, cutaneous leishmaniasis (CL) is one of the most important vector borne diseases in Iran (1) and still the leading cause of considerable morbidity of a large number of people in the endemic foci characterized by chronic skin lesions followed by scars and deformation of the infected tissue (2).

*Phelebotomus papatasi* is the main and proven vector and *Leishmania* (*Leishmania*) *major* is the causative agent of Zoonotic CL in Iran. *Leishmania major*, *L.* (*L*.) *tropica* and *L.* (*L.*) *aethiopica* cause CL in the Old World (3). The parasite has been isolated and identified from naturally infected *P. papatasi*, *P. caucasicus*, *Rhombomys opimus, Meriones libycus* and human in such endemic areas (4–8). The *P. papatasi* and *M. libycus* were as proven vector and reservoir in Qomrood, Iran district (9) and as probable vector and reservoir in Ghanavat district (10). In addition, a big part of the lowland areas of Qom Province provides good ecological niches for *P. papatasi* and therefore it has higher transmission potential (11).

In the recent years, molecular methods have been employed for identification of certain species of *Leishmania*, either isolated from cultures or from patients (3) as well as in the detection of the parasite in individual or pooled Phlebotomine specimens (12). Successful establishment of the disease in an endemic area is the outcome of a close association between the *Leishmania* parasite and its natural sand fly vector (13). Thus, vector and parasite identification have great impact on predicting expansions of the disease in endemic area, also help authorities to design new strategic programs to limit spreading vectors and disease (14, 15).

Molecular methods are increasingly employed for diagnostic and epidemiological purposes in order to confirm *Leishmania* infection and to characterize the parasites at the species or genotype level in hosts and vectors (16, 17). The detection of *Leishmania* parasites by PCR methods is highly specific and sensitive, with values reaching up to 100%. Accurate and sensitive diagnostic and identification procedures are required to distinguish *Leishmania* species/strains whose geographic distribution can overlap, which is crucial for adequate treatment and appropriate public health control measures (18).

Based on a rapid increase in incidence of CL reported in an endemic area of Kahak District of Qom Province in central Iran (19), and due to lack of knowledge on the epidemiological situation of CL, this study was conducted to determine the epidemiological features of CL including human infection and the reservoir hosts and their putative vector species in this endemic area during 2015.

## Materials and Methods

### Study area

**Fig. 1.**
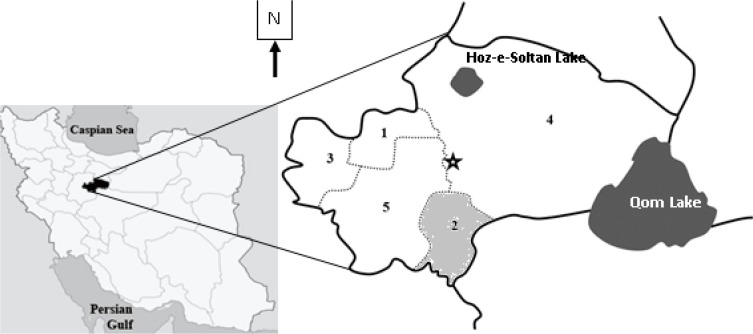
Map of Iran and Qom Province, highlighting the location of Kahak District, (2) within Qom Province (22, 23)

The Qom Province is bounded by Tehran Province in the north, Esfahan Province in the south, Semnan Province in the east, and Markazi Province in the west with an area of approximately 11240 square kilometers (0.68% total area of Iran) (Fig. 1). This study was performed from Apr to Nov 2015 in 3 villages (Khor Abad, Sarm, and Ghobadbezan) of Kahak rural district (34°09′–35°11′ N latitude and 50°06′–51°58′ E longitude) of Qom Province with the elevation of almost 1500m above sea level (20). The average annual minimum and maximum temperatures were 16.5 °C and 49 °C in Jan and Jul, respectively. The total annual rainfall was about 150mm and the average Max and Min monthly Relative humidity were 84% and 28% in Dec and Jun, respectively (21).

### Sand fly collection

Sand flies were collected from indoor (bedroom, bathroom, toilets, hall, and stables) and outdoor (rodent burrows) fixed places from the first half of Apr 2015 to the first half of Nov 2015. To capture the sand flies, 30 Sticky Paper Traps (castor oil coated white paper 20×32cm) was used twice a month from sunset to sunrise. The caught sand flies were transferred to the laboratory in Qom Health Center. To species identification, the head and the last two abdominal segments of the sand flies were mounted in Puris’ medium (24) and identified after 24–72h, using the morphological characters (25). Then, they were counted and segregated by sex. The rest of the abdomen, the wings and the legs used for DNA extraction were stored in 1.5ml sterile micro tubes containing 96% ethanol. The females were examined for abdominal status and the numbers of unfed, fresh-blood fed, and gravid and semi-gravid sand flies were recorded. The parous females were distinguished from nulliparous sand flies by observation of the appearance of the accessory glands (26). In order to determine the natural promastigote infections of female sand flies, some unfed, blood fed, semi-gravid and gravid female sand flies (captured from rodent burrows) dissected in a fresh drop of sterile saline (9/1000) for the presence of promastigotes in alimentary canal during Jun and Sep 2015.

### Reservoirs collection and smear preparation

**Fig. 2.**
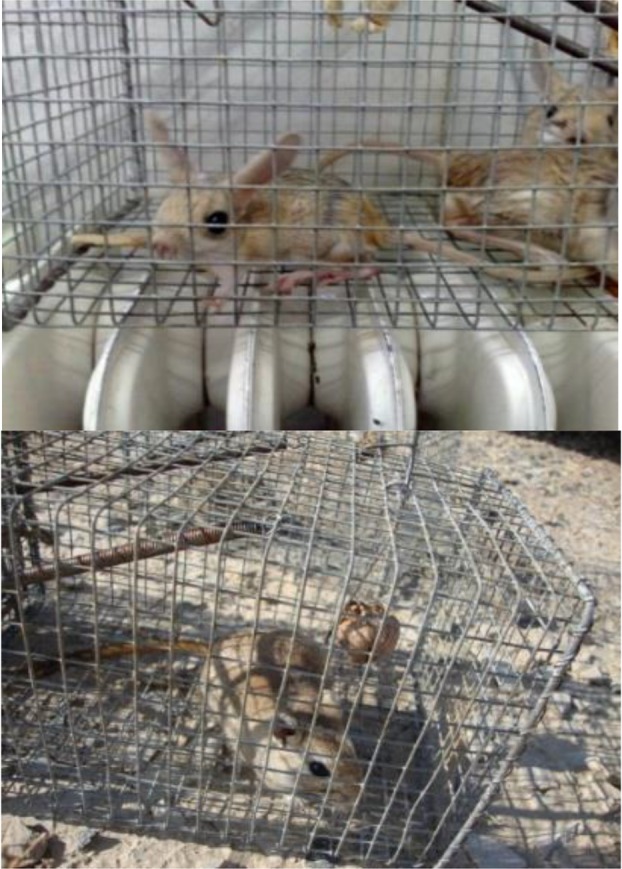
Specimens of some collected rodents in Kahak district, Qom Province, 2015

The rodents were captured by Sherman Live Traps (Fig. 2). Collection of dipodids (Rodentia: Dipodidae) was done by using small insect nets. Then the impression smears of ears were fixed with absolute methanol and examined by Giemsa staining method. The Leishman bodies were observed using light microscope and then were subjected to molecular technique for identification. Tissue samples were also taken from the edge of the lesions in 45-suspected CL patients. Lesion smears were fixed with absolute methanol and then stained with Giemsa for CL diagnosis. In this present study, we also calculated the relative frequency of CL.

### DNA extraction

DNA extractions were carried out by crashing of the contents on the slides prepared from the digestive system of the 180 dissected female sand flies, the 78 rodent’s ear lobes serous fluids and the 45 patient’s lesion tissues, using the Bioneer Genomic DNA Extraction Kit. The extracted DNA was stored −20 °C for long storing and at 4 °C for daily working. DNA from *L. major* (MRHO/IR/75/ER) that provided by Department of Medical Parasitology, School of Public Health, Tehran University of Medical Sciences (TUMS), Iran was used as positive control. Total DNA was extracted from the smears by digestion in 100 μl PBS buffer and the tube was centrifuged at 10000rpm for 10min, then 300μl lysis buffers and 30μl proteinase K added. The tube was incubated for 24h at 37 °C before adding 300μl saccharin phenol. After adding this solution, the tube was centrifuged at 9300 rpm for 5min. After transferring upper phase to new tube, 300μl phenol-chloroform should be added and was centrifuged at 10000rpm for 5min. Again transferred the upper phase to new tube and washed with pure chloroform. Thirty μl MgCl_2_ and 1000μl ethanol were added to upper phase and stored at −20 °C for 2h before was centrifuged at 10000rpm for 10min and washed down phase by 70% ethanol with TE and was centrifuged at 10000 rpm for 10min and the TE buffer was added.

### Polymerase chain reaction-restriction fragment length polymorphism (PCR-RFLP) for detection of *Leishmania* infection

The DNA samples were examined for the *Leishmania* species ITS1 by PCR amplification using the universal primer pair L5 8S (5′-TgA-TaC-CAC-TTA-TCg-CAC-T-<T>-3′) and LITSR (5′-CTg-gAT-CAT-TTT-CCg-AT-<g>-3′) (Table 1).

**Table 1.** The sequence of primers used in PCR amplification the first internal transcribed spacer (ITS1)

**Primers**	**The sequence**	**Response function**	
LITSR	5′CTGGATCATTTTCCGATG3′	Forward	**18bp**

L5 8S	5′TGATACCACTTATCGCACTT3′	Reverse	**20bp**

### Molecular study

PCR production was followed by RFLP technique (27). The cycling conditions were 95 °C for 5min., followed by 35 amplification cycles, each consisting of three steps: denaturation at 94 °C for 30sec, annealing 48 °C for 30sec, and extension at 72 °C for one min, followed by a final extension at 72 °C for 7min in thermocycler.

PCR productions were digested with the restriction endonuclease Hae III for 2 h at 37 °C. The restriction fragments were separated by electrophoresis on agarose gel and compared with those of standard reference strain of *L. major* and negative control (distilled water).

## Results

A total of 4164 sand flies (68.90% from outdoors and 31.10% from indoor resting places) were collected using sticky paper traps. Two genera (*Phlebotomus* and *Sergentomyia*) and 14 species were identified, including *Phlebotomus* (*Phlebotomus*) *papatasi* (61.74%), *P.* (*Paraphlebotomus*) *sergenti* (6.87%), *P*. (*Paraphlebotomus*) *alexandri* (1.00%), *P.* (*Paraphlebotomus*) *caucasicus* (2.55%), *P.* (*Paraphlebotomus*) *caucasicus* group (4.13%), *P*. (*Larroussius*) *kandelakii* (5.72%), *P*. (*Larroussius*) *tobbi* (0.10%), *P*. (*Larroussius*) *major* (5.62%), *P*. (*Adlerius*) *halepensis* (0.10%), *P*. (*Adlerius*) *brevis* (0.05%), *P. adlerius* group (0.31%), *Sergentomyia* (*Sergentomyia*) *sintoni* (10.83%), *S*. (*Sergentomyia*) *theodori* (0.91%) and *S*. (*Rondanomyia*) *pawlowskii* (0.07%) (Table 2).

**Table 2.** The fauna and the number of collected sand flies from the endemic area in Kahak District, Qom Province, 2015

**Site**	**Outdoor**		**Indoor**		**Total**					**Each species of the total**

**Species**	**Male**	**Female**	**Male**	**Female**	**Outdoor**	**Indoor**	**Male**	**Female**	**Total**	**%**

	**No. (%)**	**No. (%)**	**No. (%)**	**No. (%)**	**No. (%)**	**No. (%)**	**No. (%)**	**No. (%)**	**No. (%)**	
***P. papatasi***	915 (45.57)	1093 (54.43)	216 (38.37)	347 (61.63)	2008 (78.10)	563 (21.90)	1131 (43.99)	1440 (56.1)	2571 (100)	61.74
***P. sergenti***	41 (40.20)	61 (59.80)	131 (71.20)	53 (28.80)	102 (35.66)	184 (64.34)	172 (60.14)	114 (39.86)	286 (100)	6.87
***P. alexandri***	7 (58.33)	5 (41.67)	11 (36.67)	19 (63.33)	12 (28.57)	30 (71.43)	18 (42.86)	24 (57.14)	42 (100)	1.00
***P. caucasicus***	45 (100)	…	61 (100)	…	45 (42.45)	61 (57.55)	106 (100)	…	106 (100)	2.55
***P. caucasicus group***	…	157 (100)	…	15 (100)	157 (91.28)	15 (8.72)	…	172 (100)	172 (100)	4.13
***P. kandelakii***	23 (31.51)	50 (68.49)	35 (21.21)	130 (78.79)	73 (30.67)	165 (69.33)	58 (24.37)	180 (75.63)	238 (100)	5.72
***P. tobbi***	1 (100)	0 (0)	0 (0)	3 (100)	1 (25.00)	3 (75.00)	1 (25.00)	3 (75.00)	4 (100)	0.10
***P. major***	6 (24.00)	19 (76.00)	60 (28.71)	149 (71.29)	25 (10.68)	209 (89.32)	66 (28.21)	168 (71.79)	234 (100)	5.62
***P. halepensis***	0 (0)	…	4 (100)	…	0 (0)	4 (100)	4 (100)	…	4 (100)	0.10
***P. brevis***	1 (100)	…	1 (100)	…	1 (50.00)	1 (50.00)	2 (100)	…	2 (100)	0.05
***P. adlerius group***	…	2 (100)	…	11 (100)	2 (15.38)	11 (84.62)	…	13 (100)	13 (100)	0.31
***S. sintoni***	207 (50.36)	204 (49.64)	18 (45.00)	22 (55.00)	411 (91.13)	40 (8.87)	225 (49.89)	226 (50.11)	451 (100)	10.83
***S. theodori***	15 (51.72)	14 (48.28)	6 (66.67)	3 (33.33)	29 (76.32)	9 (23.68)	21 (55.26)	17 (44.74)	38 (100)	0.91
***S. pawlowski***	0 (0)	3 (100)	0 (0)	0 (0)	3 (100)	0 (0)	0 (0)	3 (100)	3 (100)	0.07
**Total**	1261 (43.93)	1608 56.07)	543 (41.92)	752 (58.08)	2869 (68.90)	1295 (31.10)	1813 (43.54)	2351 (56.46)	4164 (100)	100

Two species of *P. halepensis* and the *S. pawlowskii* were not captured from outdoor and indoor respectively. In the rodent burrows, *P. papatasi* appeared nearly May and disappeared in the late Oct and in indoors, it appeared in the late Jun and disappeared in the early Oct.

**Fig. 3.**
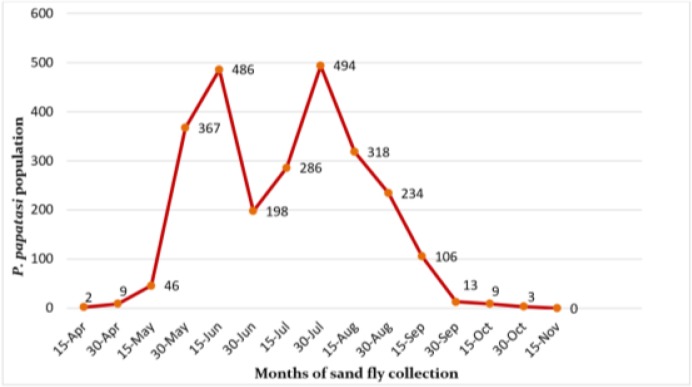
The month wise density of *Phlebotomus papatasi* in Kahak District, Qom Province, 2015

The month wise density of *P. papatasi* in rodent burrows and indoors are shown in Fig. 3. The peak of activity was detected for *P. papatasi* first in the late Jun and the second in the early Aug (Fig. 3).

The sex ratio calculates at 83.71 and 62.25 in outdoors and indoors for *P. papatasi* respectively as well as 101.5 in outdoors for *S. sintoni*. In Aug and Sep 2015 a total of 211 sand flies were collected in the vicinity of rodent burrows including *P. papatasi* (51.66 %), *P. caucasicus* (10.9%), *P. sergenti* (18%), and *S. sintoni* (19.44%).

Two percent of *P. papatasi* species had promastigote infections. The total of 300 *P. papatasi* specimens was selected and dissected for *Leishmania* infection, the result showed 67.33%, and 32.67% dissected sand flies were parous and nulli parous, respectively (Table 3).

**Table 3.** Natural infection of sand flies with promastigote, Kahak District, Qom Province, 2015

**Species**	**Catch place**				**Physiological status**								**Age Group**				**Total**	**Infected specimens**	

	I*		O**		**Unfed**		**Blood fed**		**Semi gravid**		**Gravid**		**Parous**		**Nuli parous**				
	
	**No**	**%**	**No**	**%**	**No**	**%**	**No**	**%**	**No**	**%**	**No.**	**%**	**No.**	**%**	**No.**	**%**		**No.**	**%**
***P. papatasi***	150	50	150	50	180	60	60	20	20	6.67	40	13.33	202	67.33	98	32.67	300 (100)	6	2%
***P. sergenti***	20	50	20	50	20	50	20	50	0	0	0	0	31	77.50	9	22.50	40 (100)	0	0
***P.caucasicus***	10	50	10	50	20	100	0	0	0	0	0	0	18	90	2	10	20 (100)	0	0

*: Indoor,

**: Outdoor

**Fig. 4.**
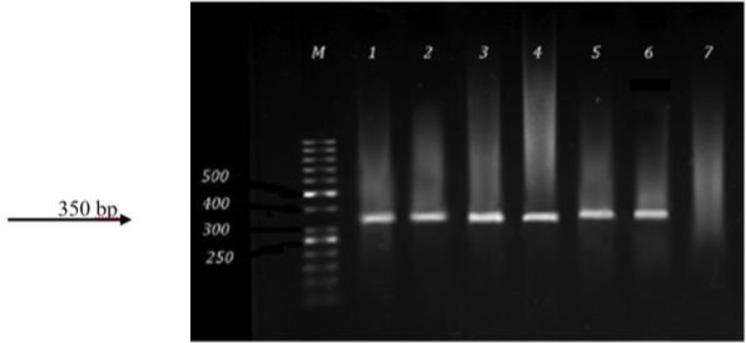
Agarose gel electrophoresis of the first internal transcribed spacer (ITS1) - Polymerase chain reaction (PCR) products. M, 50bp ladder: lane 1: *Leishmania major* (MRHO/IR/75/ER), 2, 3: *Leishmania major* in human skin samples, lane 4, 5, 6 *Leishmania major* in *P. papatasi*, N: negative control (distilled water), M: Marker

**Fig. 5.**
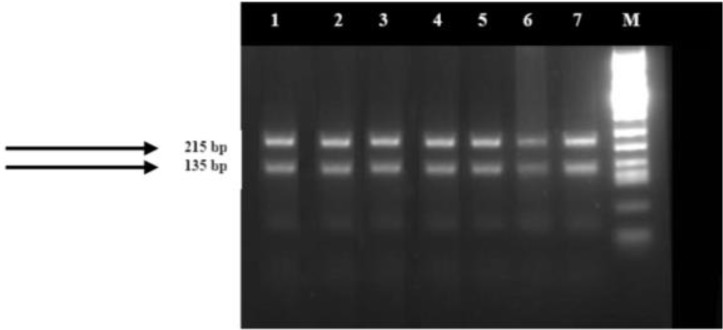
Agarose gel electrophoresis of Polymerase chain reaction-restriction fragment length polymorphism (HaeIII) products. M, 50bp ladder: lane 1, 2, 3, 4, 5, 6 *Leishmania major* in *P. papatasi*, lane 7 positive *Leishmania major* (MRHO/IR/75/ER), M: Marker

The analysis of physiological status of dissected *P. papatasi* revealed (60%) unfed, (20%) blood-fed, (6.67%) semi-gravid and (13.33%) gravid (Table 3). The used *Leishmania* primers of the PCR technique successfully amplified the ITS1 region of the strains *L. major* and *L. tropica* revealed that among 360 specimens sand flies examined by PCR, 6 parous specimens (2%) were infected by *L. major* (Fig. 4, 5).

**Fig. 6.**
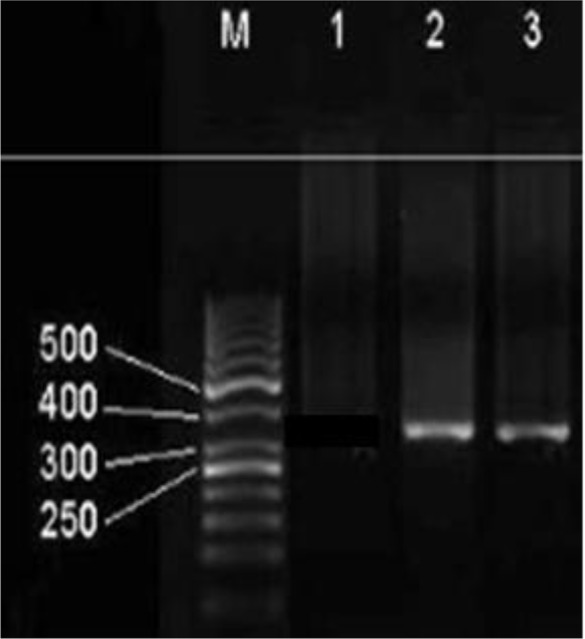
Agarose gel electrophoresis of the first internal transcribed spacer (ITS1) - Polymerase chain reaction (PCR) products. M, 50bp ladder: lane 1, negative control (distilled water), lane 2, *Leishmania major* (MRHO/IR/75/ER), lane 3 *Leishmania major* isolated from *Meriones libycus*, M: Marker

Among 92 collected rodents (67.39%), (8.70%), (4.35%), (17.39%) and (2.17%) were identified as *M. libycus*, *Nesokia indica*, *Mus musculus*, *Allactaga elater* and *Hemiechinus auritis* respectively (Table 4). *Leishmania major* has been detected in 2 (3.23%) out of 62 *M. libycus* by PCR (Fig. 6). Both of the infected *M. libycus* were females.

**Table 4.** Molecular detection of *Leishmania* infection in rodents, Kahak District, Qom Province, 2015

**Species**	**No. Rodents**		**No. exanimated**		**No. infected**	**Infected rate, % (ITS1-PCR)**

	**No**	**%**	**No**	**%**		
***Meriones libycus***	62	67.39	62	100	2	3.23
***Nesokia indica***	8	8.70	8	100	0	0
***Mus musculus***	4	4.35	4	100	0	0
***Allactaga elater***	16	17.39	4	25	0	0
***Hemiechinus auritis***	2	2.17	0	0	0	0
**Total**	92	100	78	84.78	2	2.17

All of the 45 human cases, which examined through observation passively, confirmed by PCR but only 15 numbers of them were positive by light microscope. In this study, the relative frequency of CL patients was 0.30%.

**Fig. 7.**
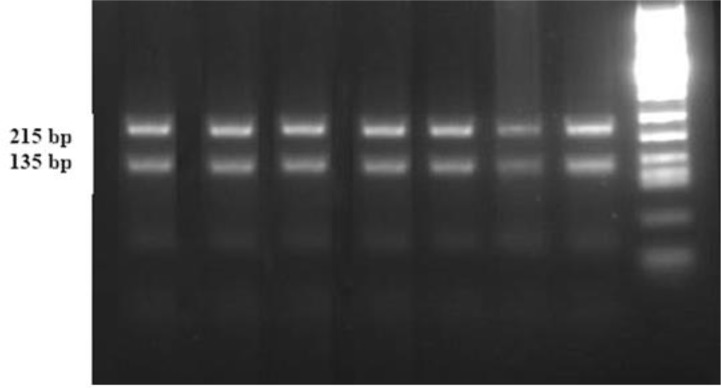
Agarose gel electrophoresis of Polymerase chain reaction-restriction fragment length polymorphism (HaeIII) analysis of ITS region for identification of *Leishmania* species using M, 50bp ladder: lane 1, 2, 3, 4 *Leishmania major* in human skin samples, lane 5, 6 *Leishmania major* isolated from *Meriones libycus*, lane 7, positive *Leishmania major* (MRHO/IR/75/ER), M: Marker

Twenty-eight (62.22%) of 45 confirmed cases were males and 17 (37.78%) were females. The mean age of patients was 30.78± 16.91. The most of the cases 34 (75.60%) occurred in autumn. Eighteen cases (40%) of patients had no positive history of traveling to leishmaniasis endemic areas during the past year. Twenty-two cases (48.89 %) of patients had one lesion. The most common location of lesion was on hands (46%). The results of PCR-RFLP indicated that 45 (100%) cases were infected as *L. major* (Fig. 7).

## Discussion

We identified *L. major* in *P. papatasi*, *M. libycus* and human by PCR technique. Previous surveys carried out in Qomrood and Ghanavat districts of Qom Province, Iran showed that *P. papatasi* and *M. libycus* were proven (in Qomrood) and probable (in Ghanavat) vector and reservoir of ZCL (9, 10). Awareness of epidemiology of ZCL is an important measure for management and planning of control (28). The entomological and reservoirs (rodents and human) fields and laboratory surveys accompanied by correct identification the common agent in their target part of their bodies is a major component for combating against disease.

Recently, genetic analyses and genotyping using PCR-RFLP have been applied and performed on vector (s) and reservoir hosts of ZCL (29). This paper reviews recent advances on sand flies vectors, *M. libycus* and human of ZCL, using molecular biological approaches. *P. papatasi* captured in this study is the main vector of *L. major* from animals to man in central Asia and appears to be the vector of *L. major* in Iran and 21 other countries in old world (30).

In this study, *P. alexandri* was also captured. It has been found naturally infected with promastigotes and is suspected vector of VL in Iran (31). *Phlebotomus caucasicus group*, *P. kandelaki* and *P. major* were also collected. *Phlebotomus caucasicus* was naturally infected with promastigotes in a new focus of VL in north-west of Iran (32). *Phlebotomus kandelaki* is naturally infected with *Leishmania* sp. promastigotes in northwest Iran and is suspected as probable vector of VL in the region (33). In Iran, *P. major* has been found in all area where human cases of VL have been reported and natural promastigote infection of this species has reported in endemic focus VL in Ghir County (Fars Province) South of Iran (34). Moreover, in this area, we captured *S. povlovskyi* in indoor. This result has been confirmed with the previous study in Esfahan (35). The identified species were consistent with previous study in other parts of Iran (1).

Low percentage of collected sand flies in indoor shows people prevent sand flies to enter their homes. *P. papatasi* was the predominant species of the genus *Phlebotomus* in indoors (31.10%) and outdoors (68. 90%). This suggests that *P. papatasi* is the anthropophilic species in this area. In addition, only *P. papatasi* is able to develop *L. major* in its mid gut and transfer the parasite to its proboscis to cause ZCL (36). *S. sintoni* enters the indoors because of nearby homes to rodent burrows (37). *P. major* and *P. kandelakii* are more dominant sand flies were collected from indoor after *P. papatasi* but due to the low frequency. These species do not involve in leishmaniasis transmission.

In the present study, we distinguished parity of sand flies by observation of the appearance of the accessory glands (26). Accessory glands secretions could not be as an indicator for distinguishing parous from nulliparous of *P. papatasi* females (38). In this study, the parous rate of *P. papatasi* was 67.33%, so it is not surprising to see high level of promastigote infection among this sand fly species. The same trend of infection is reported from north Siani in Egypt (39, 40). The results of dissection showed that 2% of *P. papatasi* species were naturally infected with promastigote infection. The *Leishmania* infection rate in sand flies is usually very low even in endemic areas (41). The low density of *Leishmania* parasites in *M. libycus* in this area may be due to low sensitivity of this rodent compared to *R. opimus* (4).

In the present study, based on molecular results, *L. major* was found in *M. libycus* as a reservoir, *P. papatasi* as a vector and suspected patients as incidental host. *M. libycus* as the main reservoir host of ZCL has been distributed in the northeastern, central and southwestern regions in Iran (42, 43). For the first time in 1996, isolation and characterization of *L. major* from *M. libycus* in Iran, has been reported (4). *Meriones libycus* has previously been infected with *L. major* parasites from Golestan, Esfahan and Yazd Provinces (7, 42, 44–46). *Leishmania major* was firmly identified in *M. libycus* that indicates this rodent species can be incriminated as reservoir host of ZCL in this location (47). The results of this study have shown, *M. libycus* is the main and potential reservoir host of ZCL in Kahak district of Qom Province and it has an important role in stable ZCL in this area, which has pay attention to future rodent control programs with administrative health center.

## Conclusion

This study confirms the existence of local transmission of zoonotic cycle of CL in Kahak District of Qom Province, and provides that in this cycle, *L. major* is the causative agent, *P. papatasi* is the main vector and *M. libycus* is the main reservoir of the disease.
